# Effect of curvature correction on parameters extracted from hyperspectral images

**DOI:** 10.1117/1.JBO.26.9.096003

**Published:** 2021-09-06

**Authors:** Luka Rogelj, Urban Simončič, Tadej Tomanič, Matija Jezeršek, Urban Pavlovčič, Jošt Stergar, Matija Milanič

**Affiliations:** a University of Ljubljana, Faculty of Mathematics and Physics, Ljubljana, Slovenia; b University of Ljubljana, Faculty of Mechanical Engineering, Ljubljana, Slovenia; c Jozef Stefan Institute, Ljubljana, Slovenia

**Keywords:** hyperspectral imaging, three-dimensional profilometry, Lambert cosine law, curvature correction, tissue phantom

## Abstract

**Significance:** Hyperspectral imaging (HSI) has emerged as a promising optical technique. Besides optical properties of a sample, other sample physical properties also affect the recorded images. They are significantly affected by the sample curvature and sample surface to camera distance. A correction method to reduce the artifacts is necessary to reliably extract sample properties.

**Aim:** Our aim is to correct hyperspectral images using the three-dimensional (3D) surface data and assess how the correction affects the extracted sample properties.

**Approach:** We propose the combination of HSI and 3D profilometry to correct the images using the Lambert cosine law. The feasibility of the correction method is presented first on hemispherical tissue phantoms and next on human hands before, during, and after the vascular occlusion test (VOT).

**Results:** Seven different phantoms with known optical properties were created and imaged with a hyperspectral system. The correction method worked up to 60 deg inclination angle, whereas for uncorrected images the maximum angles were 20 deg. Imaging hands before, during, and after VOT shows good agreement between the expected and extracted skin physiological parameters.

**Conclusions:** The correction method was successfully applied on the images of tissue phantoms of known optical properties and geometry and VOT. The proposed method could be applied to any reflectance optical imaging technique and should be used whenever the sample parameters need to be extracted from a curved surface sample.

## 1 Introduction

Optical imaging techniques have shown great potential in medical applications, primarily in disease diagnosis and surgical guidance.^1^ When light enters a tissue, it is affected by scattering or absorption in tissue chromophores, such as water, lipid, deoxyhemoglobin, oxyhemoglobin, and melanin.^2^ In diseased tissues, their absorption and scattering properties change, resulting in altered diffuse reflectance spectra compared with healthy tissue.^3^ Diffuse reflectance spectra can be measured by optical techniques, such as hyperspectral imaging (HSI),^4^ and quantitative information about tissue composition can be extracted from the measured images.

HSI is a spectral imaging technique capturing spectral and spatial information simultaneously.^5^ The image is a three-dimensional (3D) data cube having two spatial dimensions and one spectral dimension. It can capture hundreds of wavelength bands, including ultraviolet, visible, and near-infrared light. Regarding acquisition type, the two most popular imaging methods are scanning-based imaging and wide-field imaging. Scanning-based imaging usually generates images by acquiring the spectrum of each pixel (whiskbroom instruments) or line of pixels (pushbroom instruments), whereas the wide-field imaging usually acquires the whole scene with a two-dimensional detector in a single exposure and steps through the wavelengths.^4^ Additional information about different spectral imaging methods can be found in Ref. 6. The method has already been used in a variety of medical applications, such as diagnosis of hemorrhagic shock, detection of peripheral artery disease, and assessing the age of bruises.^1^

Optical images, including hyperspectral images, are commonly affected by the imaged object surface shape and the light–object–detector distances, resulting in undesired imaging artifacts, such as shadow regions.^7^ In HSI, the spectra in the affected regions are thus significantly altered, making the image analysis in these regions inaccurate. To eliminate the artifacts, the imaging setup must be improved or appropriate preprocessing of the images performed. The imaging setup can be improved by providing isotropic homogeneous illumination of the whole object, which can be realized by dome illumination, where the dome is covered by a highly reflective layer. This approach is commonly used in laboratory setups when imaging food samples.^8^ However, this approach is unsuitable for the majority of applications because it significantly limits the size of the imaged objects and requires the object to be located within the illumination dome. Moreover, it can also introduce artifacts in the recorded spectra due to multiple reflections of light from the object and the dome.

The preprocessing algorithms for the elimination of the artifacts require additional information about the object shape, illumination, and imaging distances. In the biomedical optics field, such algorithms were realized for the spatial frequency-domain imaging (SFDI) method. SFDI method projects specific illumination patterns on imaged objects, allowing the extraction of optical properties.^9^ Since the projected patterns are altered due to the object surface shape, the recorded images of the patterns also include information about the surface shape, and thus the object surface shape can be simultaneously extracted. Gioux et al.^9^ reported the image correction algorithm using Lambert cosine law and the surface shape information obtained from the phase-shifted profilometry. The imaging system was initially calibrated using a flat phantom imaged at different heights and angles to obtain necessary calibration factors. They obtained improved images of objects in regions where the surface inclination angle was lower than 40 deg and the object–camera distance is less than 3 cm. Their approach was improved by Zhao et al.^10^ by adding a correction factor, which empirically accounts for the interobject diffuse reflectance, to the Lambert cosine correction. The modified correction algorithm was tested on hemispherical tissue phantoms of known optical properties. They demonstrated that by modifying the Lambert cosine law, the maximum acceptable inclination angle increased to ∼70  deg on hemispherical objects. Van de Giessen et al.^11^ implemented Gioux’s method^12^ in a snapshot imaging system resulting in almost instantaneous capture of the sample absorption and scattering coefficients. No information about the maximum correction angle was provided. In a recent study, Gevaux et al.^13^ reported using a combined liquid crystal tunable filter HSI system and phase-shift profilometry for imaging human faces. The system recorded an image with 31 different wavelengths within 5 s. The 3D chromophore concentration maps of the faces were obtained, while the image correction was not applied, although the surface shape information was available. Therefore, the artifacts were present in the inclined and more distant regions of the chromophore maps.

In this work, the effect of the surface curvature and distance correction algorithm on sample properties extracted from the images obtained by a pushbroom HSI system combined with a 3D laser profilometer is studied. The common hyperspectral image analysis pipeline^4^ was augmented by the Lambert cosine and height correction. The results of the correction method applied to images of different objects (a hemisphere, a LEGO figure, and a human finger) with arbitrary shapes were published in 2019,^14^ but without extracting the object parameters. In that study, it was shown that the method successfully corrects the reflectance images obtained at 650 nm for surfaces where the inclination angle is <70  deg. However, this result does not provide information about the effect of the image correction on the sample properties extracted from the images. The main goal of this study was thus to assess the effect of the curvature and distance correction on the extracted biological tissue properties, also considering the actual optical properties of the samples. Therefore, tissue phantoms of known optical properties and hemispherical shape were prepared and imaged. The recorded images were corrected and phantom parameters were extracted from the corrected and uncorrected images using the inverse adding–doubling (IAD) method. In addition, the correction method was also tested on images of human hands during the cuff test. The extracted parameters from the uncorrected and corrected spectra were compared. The image correction method is expected to improve the accuracy of the extracted sample properties.

## 2 Materials and Methods

### 2.1 Hyperspectral System with 3D Profilometry Module

The HSI system was a custom-build pushbroom system. The core of the system is an imaging spectrograph ImSpector V10e (Specim, Finland) with a slit size of 30  μm. The spectrograph has no keystone distortions meaning that no additional corrections are needed. For image acquisition, a CMOS camera (Blackfly S BFS-U3-51S5M-c, FLIR Integrated Imaging Solutions, Canada) is mounted on the top of the spectrograph. The camera has a resolution of 2448×2048  pixels (5 MP) and a detector size of 2/3”. The specified pixel size of the detector is 3.45  μm. The imaging lens is a 17-mm Xenoplan 1.4/17-0903 (Scheneider, Kreuznach, Germany) resulting in the imaging line size of 14±1  cm. The working distance of the system is 30 cm. The longitudinal length of the image is defined by selecting the number of imaged lines and was in this study 5 cm. The hyperspectral camera is mounted on a translational stage (8MO195X-340-2.5, Standa, Lithuania). The stage is connected to the computer via a USB controller (8SMC4-USB-B8-1, Standa, Lithuania), enabling scanning control.

**Fig. 1:**
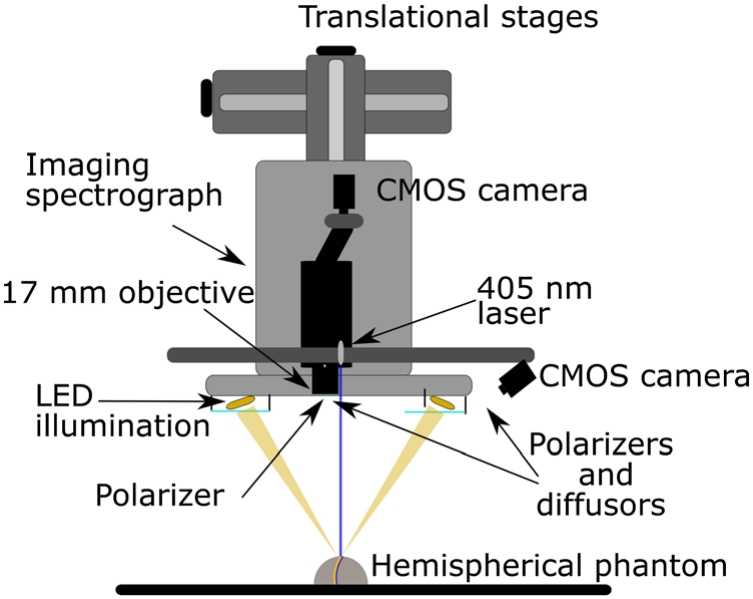
Schematics of an HSI system with 3DP.

Imaging was performed in reflectance mode. A custom-made LED illumination system was developed. The illumination is composed of four LED panels distributed symmetrically across the scanning line, as shown in Fig. 1. In each panel, two types of LEDs are used. In the inner panels, 10 white LEDs (LCW H9GP, Oslon Black Warm White, Osram, Germany) and 10 780-nm LEDs (SMB1N-780D, Roithner Lasertechnick, Germany) were mounted periodically (one white and one 780 nm). In the outer panels, 10 850-nm (SFH 4715S, Olson Black, Osram, Germany) and 10 950-nm (SFH 4725S, Olson Black, Osram, Germany) LEDs were mounted in a similar way. Combination of all LEDs covered continuously the wavelength range 400 to 1000 nm. By adding a thin diffuser in front of the panels, homogeneous illumination of the sample was obtained. To achieve illumination parallel to the optical axis of the camera, all the panels were carefully symmetrically aligned around the camera objective. To assure thermalization of the illumination system, water cooling system was developed. Thermalization was necessary because the change of the LED temperature changes its emission spectrum and consequentially alters the measured sample reflectance. More details about the illumination can be found in Ref. 15. Two-wire grid polarizers (Bolder Vision Optik) were fixed in front of the objective and LED panels in the cross-polarized configuration to eliminate specular reflectance. The spectral resolution of the system is 0.5 nm, and spatial resolution is 0.3 mm.

The recorded raw spectra are converted to the normalized reflectance spectrum $I_{ij}$ at wavelength λ using the following equation:

$$I_{ij}(\lambda )=\frac{R_{ij}(\lambda )-D_{ij}}{W_{ij}(\lambda )-D_{ij}},$$ (1)

where $R_{ij}$ stands for the raw measured sample spectrum at image position (i,j), $D_{ij}$ is the dark current at the same point and integration time, and $W_{ij}$ is the spectrum of a white standard. The white standard was Spectralon (Labsphere Inc., New Hampton), reflecting 99.9% of the light.

The hyperspectral system is combined with a 3D profilometry (3DP) module. It is composed of a laser projector (FLEXPOINT, 30 mW, 405 nm) and a monochromatic camera. The laser line is parallel to the hyperspectral acquisition line and has a fan angle of 65 deg and a line width of 0.3 mm. The offset between the laser line and hyperspectral system is 1 mm to reduce laser affecting the hyperspectral image. The laser line was recorded by a monochromatic camera (Flea3, FL3-U3-13Y3M-C, FLIR, Canada) with a resolution of 1280×1024  pixels. To detect the laser line, a 405-nm bandpass filter was used. The camera is positioned at a 26-deg angle regarding the laser plane to get sufficient Z-axis resolution. The module is based on the laser line triangulation technique. The laser projects a thin line onto the object during scanning. The line is distorted due to the sample shape, and the line image is captured by the monochromatic camera. From the distorted line image, the surface shape is calculated using the following equation:

$$\Delta Z=\frac{L}{\tan(\beta )+\tan(\alpha -\arctan(\frac{\Delta Y}{f}))},$$ (2)

where ΔZ is the height difference between two surface points, α and β are the incidence and observation angles of the 3DP module, L is the distance between the laser projector and the camera, ΔY is the laser line displacement due to the surface curvature recorded by the monochromatic camera, and f is the imaging lenses focal length (the scheme and additional explanation can be found in Ref. 14). To measure the whole sample, the surface scanning is used. In our case, the system was already mounted on the hyperspectral camera translation stage and could therefore acquire both hyperspectral and 3D images at the same time.

3DP system was calibrated using a custom-build reference object of known geometry. The estimated resolution of the system is 0.1 mm in the X and Y directions and 0.05 mm in the Z direction. A more detailed description of the 3DP system can be found in Ref. 16.

Due to the parallax between the 3DP camera and the laser projector, the shadowing of the laser line is present. Therefore, some surface regions are not illuminated by the laser and cannot be reconstructed. To provide the complete sample surface, the missing values are interpolated. The Laplace interpolation technique was used for this purpose in our study.^17^^,^^18^

### 2.2 Lambert and Height Correction

The image correction method is described in detail in Ref. 14; therefore, only a brief overview is provided here. The distance from the camera to the sample surface varies with the sample thickness, consequently affecting irradiance detected by the camera. The height correction was performed using images of a white standard located at different distances. Mean values of the intensities measured at different distances were calculated and fitted with a quadratic function. The quadratic function was then used to correct the image values at every pixel,

$$I_{\mathrm{corr}\_h}(\lambda )=\frac{I(\lambda )}{\varepsilon (\lambda )},$$ (3)

where I(λ) represents the normalized reflectance spectrum and ε(λ) is the ratio between the fitted quadratic function at the working distance and the actual distance. The curvature artifact was corrected using the Lambert correction. The surface shape information served for the calculation of corresponding surface normals. The angle between the surface normals n and the illumination direction vectors l was calculated as

$$\cos(\theta )=\frac{n\cdot l}{n\cdot l},$$ (4)

which was later used to correct the height-corrected reflectance as

$$I_{\mathrm{corr}\_\mathrm{lam}}=\frac{I_{\mathrm{corr}\_h}}{\cos\;\theta }.$$ (5)

The described corrections were performed for the case of vertical illumination (parallel to the blue vertical line in Fig. 1) due to the absence of illumination angle effect and simpler data processing. Equations (3) to (5) were applied to every image pixel and wavelength of the normalized reflectance data cube I.

### 2.3 Tissue Phantoms

The tissue phantoms were prepared from SiliGlass according to a slightly modified recipe of Sekar et al.^19^^,^^20^ The substrate is platinum-cured silicon rubber (PlatSil^®^ SiliGlass), which is polymerized from two liquid parts, namely part A and part B. Therefore, it can reap the benefits of liquid phantoms such as easy customization of each optical layer by adding small quantities of absorber or scatterer.^21^ After combining the two parts, curing begins, and the phantom obtains the advantages of the solid phantom, such as well-defined optical properties and temporal stability. The add-ons were absorbers (Polycraft Black Silicone Pigment, MB Fibreglass, United Kingdom) and scatterers (silica microspheres, No. 440345, Sigma-Aldrich). Since the original absorber solution was very dense, it was mixed with silicone part A in ratio 1:2272 prior to adding to the phantom solution. The absorber concentrations ($C_{\mathrm{abs}}$) are provided for the diluted absorber. After determination of the necessary absorber and scatterer concentrations ($C_{\mathrm{sc}}$), the following quantities were used to calculate required masses:

$$m_{\mathrm{abs}}=(2C_{\mathrm{abs}}m_{A})/(1-2C_{\mathrm{abs}})\;m_{\mathrm{sc}}=2C_{\mathrm{sc}}(m_{A}+m_{\mathrm{abs}})\;m_{B}=m_{A}+m_{\mathrm{abs}}-m_{\mathrm{sc}},$$ (6)

where $m_{\mathrm{abs}}$, $m_{\mathrm{sc}}$, $m_{A}$, and $m_{B}$ stand for masses of the diluted absorber, scatterer, part A, and part B, respectively. Because some material remains on the cup wall the $m_{B}$ was increased by 1 g, which was deduced experimentally. The $m_{\mathrm{sc}}$ was increased accordingly to obtain the desired scatterer concentration.

Here, only a brief description of the phantom preparation is provided. An interested reader can find more information in Refs. 19 and 20. First, the absorber and part A were mixed in a disposable plastic cup for 10 min using a magnetic stirrer and a glass rod. The mixture was put into an ultrasound bath (ASonic PRO 08, Slovenia) for 10 min and into four millibar vacuum chamber for 10 min to eliminate bubbles formed during the stirring process. At the same time, the second part of the phantom was prepared. The raw microspheres were mixed by glass rod and shaken by hand to break the large clusters. The prepared microspheres were mixed with part B. The mixture was stirred with the magnetic stirrer for 10 min, ultrasonicated for 10 min, and vacuumed for 10 min. To perform the following steps, the polymerization was slowed down by cooling the mixtures to 15°C. During the cooling, the microspheres sedimentation occurred; therefore, the mixtures were mixed again with a magnetic stirrer for 3 min and vacuumed for 5 min. Part B mixture was added to part A and mixed using the glass rod and magnetic stirrer for 3 min and degassed in the vacuum chamber for 8 min. The final blend was poured in a Teflon cake pops tray with hemispherical shapes and left in a 25°C water bath. The water bath was necessary to speed up the polymerization to prevent the miscrospheres sedimentation. A thin layer of transparent SiliGlass was formed on the surface of the phantom, affecting the optical properties. Therefore, the phantom surface was sanded using fine sandpaper as the final step of the preparation.

Seven different tissue phantoms with absorber concentrations $C_{\mathrm{abs}}=3.6\%$ to 37% and scattering concentrations $C_{\mathrm{sc}}=1.6\%$ to 6.5% following the recipe^19^ were prepared. The selected concentration ranges correspond to absorption coefficients of 0.1 to $1.1\;{\mathrm{cm}}^{-1}$ and reduced scattering coefficients of 8 to $40\;{\mathrm{cm}}^{-1}$. The complete spectra are presented in Appendix A. According to Bashkatov et al.^22^ these properties are within the ranges reported for skin, subdermis, and muscles.

The refractive index of the phantoms was reported in Ref. 20:

$$n^{2}(\lambda_{r})=1+\frac{0.95007197\lambda_{r}^{2}}{\lambda_{r}^{2}-0.05943376},\;\lambda_{r}=\frac{\lambda }{436.4\;\mathrm{nm}}.$$ (7)

**Fig. 2:**
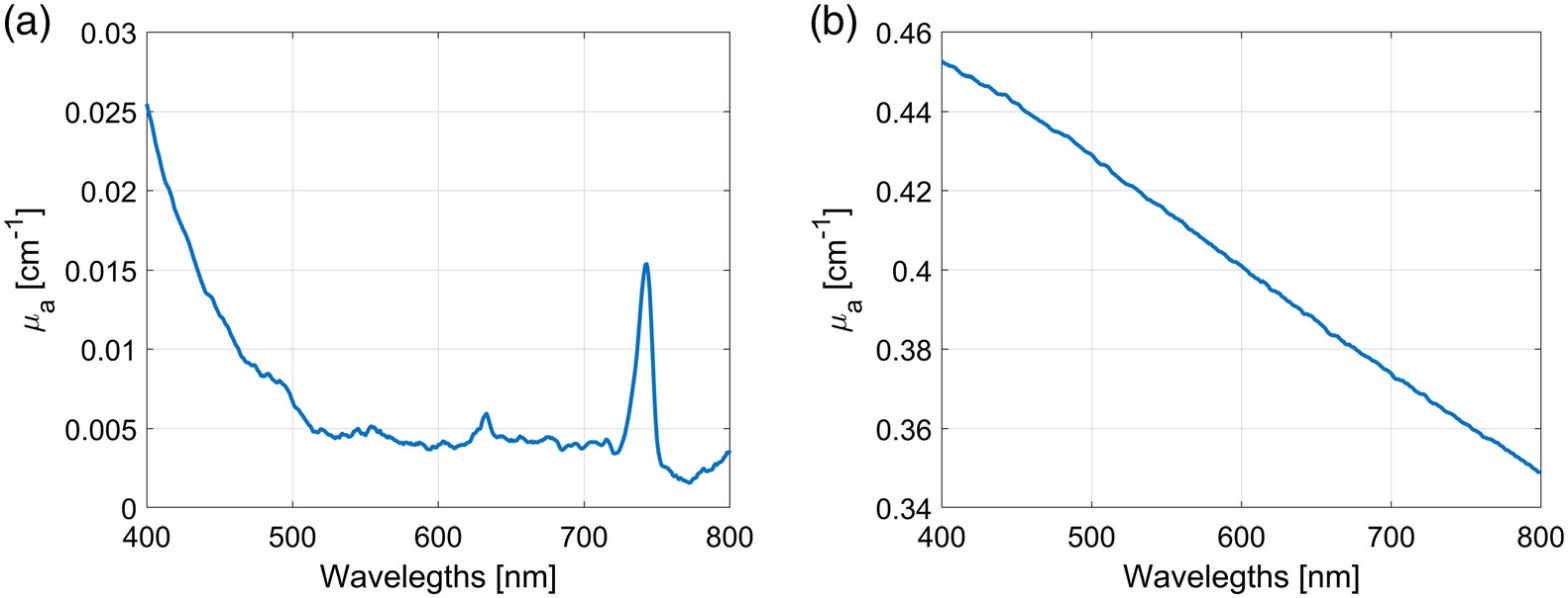
Absorption coefficients of (a) clear polymerized SiliGlass and (b) pigment with $5.83\times 10^{-5}$ mass fraction.

The absorption coefficients of the phantoms depend on the pigment concentration $C_{\mathrm{abs}}$ and the absorption of the SiliGlass medium. Transmissions of the transparent SiliGlass medium and the pigment were measured on a transmission spectrometer (Lambda 960, Perkin-Elmer). Taking into account the path length and multiple reflections on the 1-cm polystyrene cuvettes, the absorption coefficients were calculated from the transmissions. The absorption coefficients of the medium and the absorber with the mass fraction $5.83\cdot 10^{-5}$ are presented in Fig. 2.

**Fig. 3:**
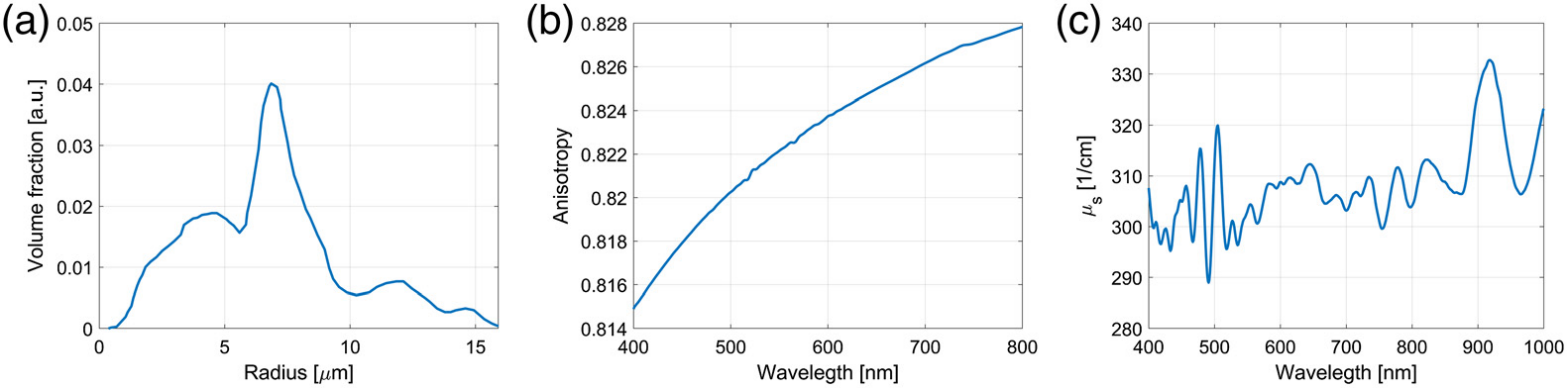
Volume fraction of (a) sphere distribution, (b) anisotropy, and (c) scattering coefficient as a function of wavelength.

The scattering coefficient and anisotropy factor g of the phantoms were calculated using Mie theory and measured volume fraction distribution of the microspheres [Fig. 3(a)]. The volume fraction of the spheres was determined experimentally by imaging multiple samples of the microspheres by SEM (FEI HeliosNanolab 650) and analyzing the obtained SEM images. Evidently, the distribution has a peak at ∼7  μm radius, with spheres radii ranging from 1 to 16  μm. The Mie calculations were performed according to Ref. 23 for microspheres of different radii and the shell thickness of 1  μm. The results of the Mie calculation were scattering coefficients and anisotropy factors. The scattering coefficients and the anisotropy factors for single radius, and the volume fraction distribution was used to calculate weighted averages of the scattering parameters. The average anisotropy and scattering coefficient are shown in Figs. 3(b) and 3(c).

### 2.4 Vascular Occlusion Test

The correction method was also tested on biological tissues, namely on human hand images. Five healthy volunteers (two males and three females) aged 23 to 25 were imaged with the hyperspectral system. The procedure was performed according to the Declaration of Helsinki. The experimental protocol was approved by the Slovenian National Medical Ethics Committee. Informed consent was obtained from the healthy subjects included in this study.

Their hands were imaged before, during, and after the vascular occlusion test (VOT) to observe hemodynamic changes. A cuff was placed on their right upper arms. The hands were placed in the HSI system with the fingers spread as much as possible to reduce the light inter-reflection between the adjacent fingers. First, the baseline image (i.e., before) of the fingers was recorded. The cuff was then inflated to over 200 mmHg to induce total blood flow occlusion. After 150 s, next image was recorded (i.e., during). Finally, the cuff was released, and the third image was acquired (i.e., after). The fingers were imaged in the region between the MCP and DIP joints. The small imaging area was chosen to prevent long imaging times.

### 2.5 Inverse Adding–Doubling and Sample Models

To extract physiological parameters from reflectance spectra, the inverse problem of light propagation in turbid media has to be solved. Models are divided into two groups of iterative and noniterative models, where iterative are most commonly used. Such methods use equations in which optical properties (absorption and scattering coefficients) are directly connected with the parameters thta are being evaluated (e.g., chromophore concentration).^22^ The same optical parameters are indirectly connected to measured parameters such as reflectance spectra. The quantities determining optical properties are iteratively changed until the measured and modeled spectra coincide with certain accuracy.^24^^,^^25^

In our research, the tissue parameters were extracted using the IAD method.^26^ IAD is a numerical technique for the extraction of optical properties of turbid media in a slab geometry. It considers the anisotropy of scattering, internal reflections from the slab boundaries, and can provide accurate results. The method is iteratively solving the one-dimensional transport equation until the calculated values of the reflectance are matched to the measured ones.^22^^,^^26^

In this research, GPU-accelerated one-layer and two-layer IAD were used on tissue phantom and human hand hyperspectral images in the spectral range 430 to 700 nm. Incoming and outcoming light was divided into 20 conical fluxes to provide the necessary accuracy. For the nonlinear least-squares fitting, the Levenberg–Marquardt algorithm was implemented on GPU with a maximum number of iterations of 200. Five hundred spectra were fitted at once with a 5-nm step. The corrected and uncorrected normalized images were first binned eight times in the spatial and six times in the spectral dimension to reduce the computational time.

The one-layer model was used to simulate light propagation in the tissue phantoms. The fitted parameters were $C_{\mathrm{abs}}$ and $C_{\mathrm{sc}}$. The layer thickness was set to 2 cm corresponding to the thickness of tissue phantoms. The optical parameters of the phantom components were presented in the previous section. The absorption coefficient was calculated as

$$\mu_{a}=C_{\mathrm{AB}}\mu_{\mathrm{AB}}+\frac{C_{\mathrm{abs}}\mu_{\mathrm{abs}}}{0.13246},$$ (8)

where $C_{\mathrm{AB}}$ and $C_{\mathrm{abs}}$ are the volume fractions of polymerized SiliGlass and diluted absorber, $\mu_{\mathrm{AB}}$ is the absorption coefficient of polymerized SiliGlass [Fig. 2(a)], and $\mu_{\mathrm{abs}}$ is the absorption coefficient of diluted pigment with $5.83\times 10^{-5}$ mass fraction [Fig. 2(b)]. The denominator 0.13246 is included in the absorption coefficient expression to address the mismatch between the ink dilution in case of the absorption coefficient measurement [Fig. 2(b)] and the one used for the phantoms preparation (1:2272).

The scattering coefficient was calculated as

$$\mu_{s}=C_{\mathrm{sc}}\mu_{s\_\mathrm{cal}},$$ (9)

where $C_{\mathrm{sc}}$ is the volume fraction of the scatterer and $\mu_{s\_\mathrm{cal}}$ is the calculated scattering coefficient [Fig. 3(c)]. The anisotropy factor g was extracted from the plot in Fig. 3(b).

A two-layer skin model was used to extract physiological parameters from the recorded human skin spectra. The top skin layer was a thin epidermis layer with melanin as the main chromophore, and the bottom layer was the thick dermis with blood, bilirubin, and cytochromes as absorbers.

Absorption coefficient of the epidermis is calculated using the customary relations^27^

$$\mu_{a,\mathrm{epi}}=f_{\mathrm{mel}}.\mu_{a,\mathrm{mel}}+0.25\;{\mathrm{cm}}^{-1}\;\mu_{a,\mathrm{mel}}(\lambda )=6.6\times 10^{11}\;{\mathrm{cm}}^{-1}\;{\left(\frac{\lambda }{\mathrm{nm}}\right)}^{-3.33},$$ (10)

where $\mu_{a,\mathrm{mel}}$ represents the melanin absorption coefficient and $0.25\;{\mathrm{cm}}^{-1}$ is the baseline absorption of bloodless skin according to Svaasand et al.^28^ The melanin volume fraction ($f_{\mathrm{mel}}$) is constant throughout the epidermis.

The absorption coefficient for dermis is obtained by combining the blood, the cytochrome C oxidase, and the bilirubin absorption coefficients with the baseline absorption in a manner analogous to Eq. (10):

$$\mu_{a,\mathrm{der}}=f_{\mathrm{Hb}}\cdot \mu_{a,\mathrm{Hb}}+f_{\mathrm{HbO}2}.\mu_{a,\mathrm{HbO}2}+f_{\mathrm{bil}}\cdot \mu_{a,\mathrm{bil}}+f_{\mathrm{CytO}}\cdot \mu_{a,\mathrm{CytO}}+f_{\mathrm{CytOO}2}\cdot \mu_{a,\mathrm{CytOO}2}+0.25\;{\mathrm{cm}}^{-1},$$ (11)

where $f_{\mathrm{Hb}}$ and $f_{\mathrm{HbO}2}$ are volume fractions of deoxy- and oxyhemoglobin, $\mu_{a,\mathrm{Hb}}$ and $\mu_{a,\mathrm{HbO}2}$ are corresponding absorption coefficients,^29^ $f_{\mathrm{bil}}$ and $\mu_{a,\mathrm{bil}}$ are the millimolar concentration^30^ and absorption coefficient of bilirubin, whereas $f_{\mathrm{CO}}$ and $f_{\mathrm{COO}2}$ are respective millimolar concentrations of reduced and oxidized cytochrome C oxidase and $\mu_{a,\mathrm{CO}}$ and $\mu_{a,\mathrm{COO}2}$ associated absorption coefficients.^31^ Total blood volume fraction can be calculated as the sum of $f_{\mathrm{Hb}}$ and $f_{\mathrm{HbO}2}$. Here, we report concentrations of deoxygenated and oxygenated blood as volume fractions. However, another possibility is to report the blood concentrations as millimolar concentrations. Conversion from volume percent to millimolar concentrations is straightforward and described in detail in Ref. 27.

The reduced scattering coefficient of the epidermis and dermis is described as the customary ansatz suitable for the relatively narrow spectral range used in this study:^27^

$${\mu_{s}}^{\prime }=a\cdot {\left(\frac{\lambda }{500\;\mathrm{nm}}\right)}^{-b},$$ (12)

where a represents the reduced scattering coefficient at 500 nm and b is the scattering power. The refractive index of both layers was calculated as^32^

$$n=1.309+4.36\cdot 10^{2}\lambda^{-2}+1.6065\cdot 10^{9}\lambda^{-4}-1.12811\cdot 10^{14}\lambda^{-6}.$$ (13)

The anisotropy factor was g=0.82.^33^ To improve the fitting robustness and reduce computational time, b was fixed to 1.27. In addition, the thickness of epidermis was fixed to 100  μm and the thickness of dermis to 1 cm, corresponding to a very thick tissue layer transmitting negligible amount of light.

Six of the skin parameters were the free parameters and were determined by fitting. These parameters including their lower and upper boundaries are presented in Table 1. The $f_{\mathrm{mel}}$, $f_{\mathrm{Hb}}$, and $f_{\mathrm{HbO}2}$ parameters boundary values were selected according to Verdel et al,^34^ who analyzed healthy human skin *in vivo*. The boundary values of cytochrome were taken from the publications of Bale et al.^35^ and Mason et al.^31^ The boundary values for the scattering coefficient were selected based on the values reported by Jacques^27^ and Bashkatov et al.^22^ Broader boundary intervals as expected for normal Caucasian human skin were selected to prevent forcing IAD to specific parameter values, especially in the case of the uncorrected images.

**Table 1:** Two-layer skin model parameters and corresponding boundary values.

	Minimum	Maximum
$f_{\mathrm{mel}}$ (%)	0.1	15
$f_{\mathrm{Hb}}$ (%)	0.1	20
$f_{\mathrm{HbO}2}$ (%)	0.1	20
$f_{\mathrm{CytO}}$ (mM)	0.1	2
$f_{\mathrm{CytOO}2}$ (mM)	0.1	2
a (${\mathrm{cm}}^{-1}$)	20	50

Since the extracted sample parameters can depend on the selection of the initial parameters for IAD due to the local minima, we first selected a characteristic 10×10  pixel region for each hyperspectral image. This region was fitted by selecting 1000 different randomly generated initial parameters within the specified parameter intervals (Table 1). The extracted parameters where the highest R-square value for the fitted spectra was obtained were used as the initial parameters for fitting the entire hyperspectral images. This procedure allowed us to reduce the computational time per image by ∼10 times due to faster convergence of the minimization algorithms.

## 3 Results

### 3.1 Tissue Phantoms

**Fig. 4:**
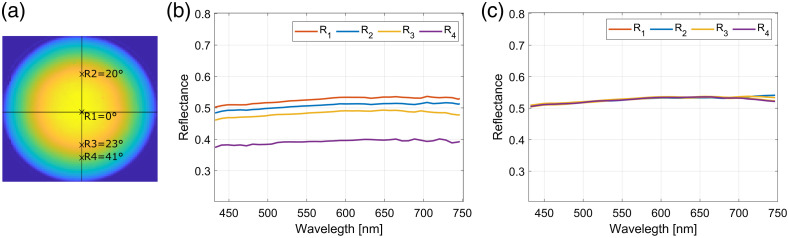
(a) An image of the hemispherical tissue phantom Aa at 600 nm. (b) The uncorrected and (c) corrected reflectance spectra from the regions R1 to R4 (see labels) of the same phantom.

When imaging a sample with spectral imaging, the inclined and more remote regions of the sample have underrated irradiance causing spectral alterations. An example of these artifacts is presented in Figs. 4(a) and 4(b) for the hemispherical tissue phantom Aa. Evidently, the more the sample surface is inclined and farther from the camera, the lower are the reflectances at all wavelengths. After the curvature and height corrections were applied to the image, the spectra in all regions became similar [Fig. 4(c)], as expected for a homogeneous phantom.

Adding–doubling algorithm was used to extract the absorber $C_{\mathrm{abs}}$ and scatterer concentrations $C_{\mathrm{sc}}$ in each pixel of the uncorrected and corrected images. The final results were constituent concentration distribution maps. The accuracy of the extraction procedure was initially examined by comparing the extracted parameters from the center of the hemisphere to the actual concentrations used in the phantoms preparation. The central region was chosen because it is relatively flat, and thus negligible spectral alterations are present. Here, the reflectance spectra are almost equal in the corrected and uncorrected images [Figs. 4(b) and 4(c)]. Tables 2 and 3 show the IAD extracted and phantom preparation concentrations of the absorbers and microspheres. For both concentrations corresponding standard deviations are also provided. The standard deviations for the preparation concentrations are the small uncertainties at different preparation steps, and local variations for the IAD extracted concentrations. Considering the standard deviations, the IAD extracted values agree very well with the concentrations used in the phantoms preparation, showing that the extraction approach is accurate when negligible artifacts are presented.

**Table 2:** Average IAD extracted concentration of the absorber $C_{\mathrm{abs}}$ in the central region of the phantoms and the concentrations according to the phantoms preparation.

Phantom	Preparation $C_{\mathrm{abs}}$ (%)	Extracted $C_{\mathrm{abs}}$ (%)	Absolute difference (%)	Relative difference (%)
Aa	3.7 ± 0.4	3.8 ± 0.4	0.1 ± 0.8	2.7 ± 0.6
Af	33.9 ± 3.5	36.9 ± 3.7	2.7 ± 7.2	7.9 ± 1.6
Cc	13.3 ± 1.3	14.3 ± 1.4	1 ± 2.7	7.5 ± 1.4
Cb	6.9 ± 0.7	7.2 ± 0.7	0.3 ± 1.4	4.3 ± 0.8
Cd	19.3 ± 1.9	21.4 ± 2.1	1.1 ± 4	5.7 ± 1.9
Df	32.1 ± 3.2	34.1 ± 3.4	2 ± 6.6	6.2 ± 1.2
Da	3.5 ± 0.4	3.6 ± 0.4	0.1± 0.7	2.9 ± 0.6

**Table 3:** Average IAD extracted concentration of the microspheres $C_{\mathrm{sc}}$ in the central region of the phantoms and the concentrations according to the phantoms preparation.

Phantom	Calculated SC (%)	Extracted SC (%)	Absolute difference (%)	Relative difference (%)
Aa	1.6 ± 0.2	1.5 ± 0.1	−0.2 ± 0.3	9.4 ± 2.0
Af	1.6 ± 0.2	1.4 ± 0.1	−0.2 ± 0.3	12.5 ± 2.8
Cc	5.2 ± 0.5	4.8 ± 0.5	−0.4 ± 1.0	7.6 ± 1.6
Cb	5.4 ± 0.5	5.1 ± 0.5	−0.3 ± 1.0	5.5 ± 1.1
Cd	5.3 ± 0.5	4.8 ± 0.5	−0.5 ± 1.0	9.4 ± 2.1
Df	7.0 ± 0.7	6.3 ± 0.6	−0.7 ± 1.3	10.0 ± 2.2
Da	7.1 ± 0.7	6.5 ± 0.7	−0.6 ± 1.4	8.5 ± 1.9

**Fig. 5:**
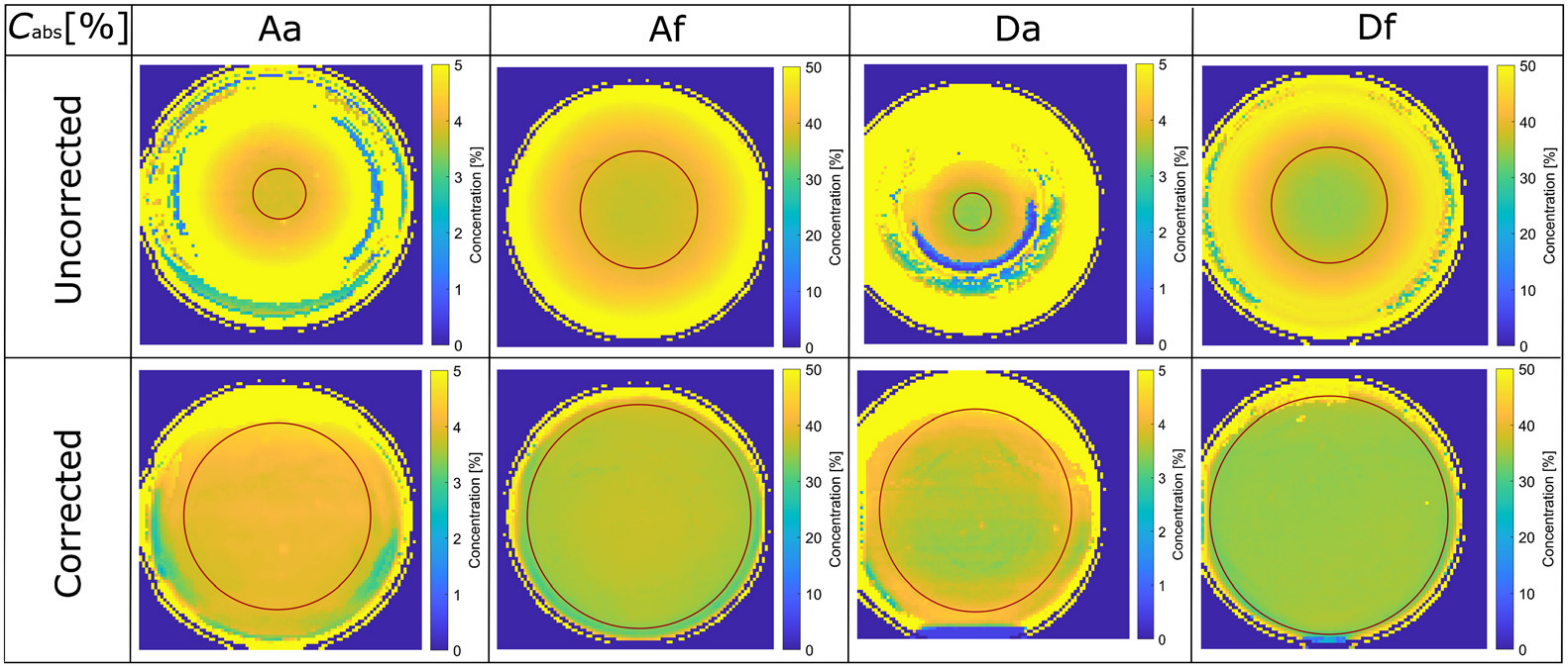
Absorption concentration ($C_{\mathrm{abs}}$) distribution maps extracted from the uncorrected and corrected images. The circles mark the region where the absorber concentrations are within 5% of the central value.

Figure 5 shows absorber concentration distribution maps for two low absorption phantoms (Aa and Da) and two high absorption phantoms (Af and Df). When the uncorrected images are analyzed (Fig. 5, first row), relatively homogeneous concentration distributions are obtained only in the central regions. The extracted values fall within the 5% interval of the accurate values (Table 2) when the inclination angle is <8±5  deg and 17±6  deg for the low and high absorption phantoms, respectively. Thus, only a tiny portion of the phantoms can be effectively analyzed. In contrast, the corrected images (Fig. 5, second row) result in much wider homogeneous regions of the phantoms. For the low absorption phantoms, the region where the extracted parameters fall within the 5% interval of the accurate values extends to the inclination angle of 50±5  deg, whereas for the high absorption phantoms, this region extends to the inclination angle of 60±3  deg. For each phantom, the inclination boundary where the extracted parameters fall within the 5% interval of the hemisphere central values is marked with a circle (Fig. 5).

**Fig. 6:**
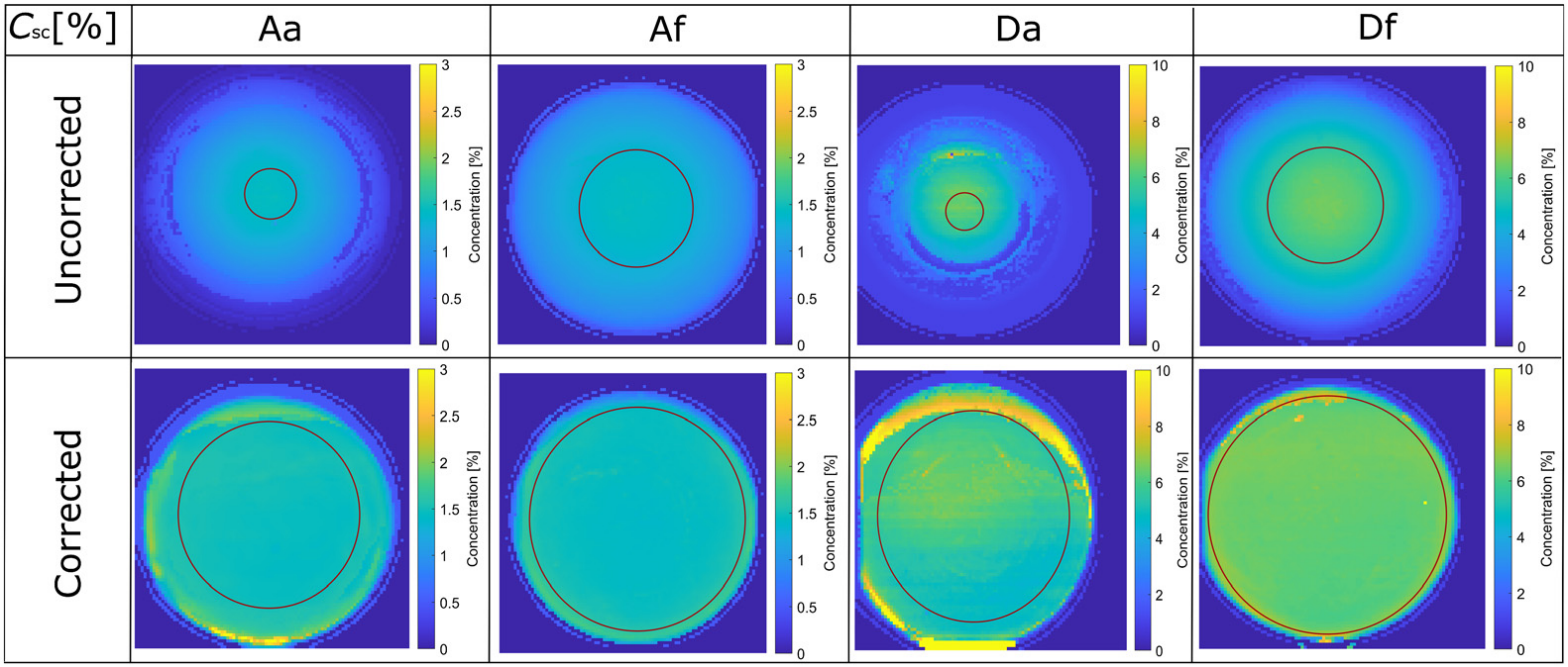
Microsphere concentration ($C_{\mathrm{sc}}$) distribution maps extracted from the uncorrected and corrected images. The circles mark the region where the scatterer concentrations are within 5% of the central value.

The extracted microspheres concentration distribution maps are presented in Fig. 6. Similar to the absorber maps, the uncorrected images (Fig. 6, top row) result in a homogeneous region only in the central part of the phantoms. The 5% region inclination angles are similar to the absorber ones. Analyzing the corrected images (Fig. 6, bottom row) yields much larger homogeneous areas. Here, the 5% region inclination angles are 49±6  deg and 58±3  deg for the low and high absorption phantoms. The inclination boundary where the extracted parameters fall within the 5% interval of the hemisphere central values is marked with a circle for each phantom (Fig. 6).

**Fig. 7:**
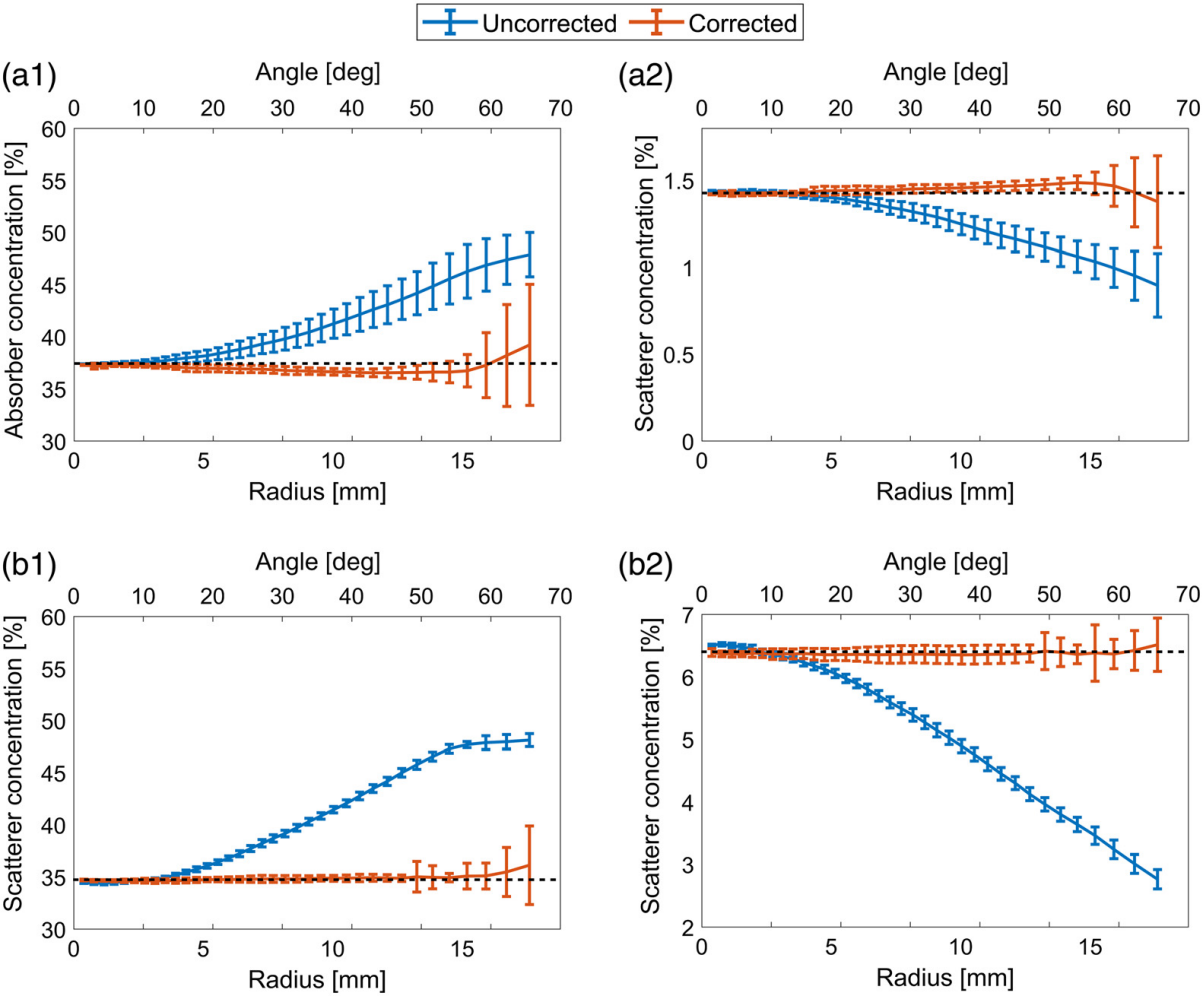
Radially averaged [(a1), (b1)] absorber and [(a2), (b2)] scatterer concentrations extracted from the corrected and uncorrected images of the phantoms [(a1), (a2)] Af and [(b1), (b2)] Df. Horizontal line in each panel represents true absorber and scatterer concentrations.

In general, the phantoms with low absorption are more affected by the artifacts than those with high absorption, whereas the scattering coefficient does not have such a significant effect on the extracted properties, at least in the range used in this study. For a more detailed view, Fig. 7 shows line plots of the extracted absorber and microspheres concentrations for the phantoms Af and Df. For these phantoms, the correction algorithm is most effective. The presented concentrations were calculated as radial averages of the maps, with zero in the hemisphere center. The standard deviations represent the radial variation of the concentrations.

Evidently, the correction significantly improves the flatness of the absorber and scattering concentrations. The correction fails to be effective close at the hemisphere boundary (∼15  mm), where the inclination angle increases above 60 deg. However, the extracted values at the boundary are still much closer to the value in the center when correction is used compared with the uncorrected image concentrations. Therefore, the correction is also valid in these boundary regions.

For every phantom, the maximum inclination angle where the concentration differs by 5% from the central value was found. This served us as a criterion for the angles at which the proposed correction successfully corrects the data. Table 4 shows the angles for all tissue phantoms. On average, the inclination angles are four times larger when the correction is used. The table also shows that the improvement is more significant when the concentration of absorbers is lower.

**Table 4:** Maximum inclination angle at which the concentrations are within 5% of the central value.

Phantom	Aa	Af	Cc	Cb	Cd	Df	Da
Corrected (deg)	47 ± 3	56 ± 4	51 ± 3	47 ± 4	51 ± 3	62 ± 5	50 ± 3
Uncorrected (deg)	8 ± 3	20 ± 5	14 ± 4	10 ± 3	18 ± 5	17 ± 4	9 ± 3

### 3.2 Human Hands

The efficacy of the correction method was tested on human fingers imaged before, during, and after VOT.

**Fig. 8:**
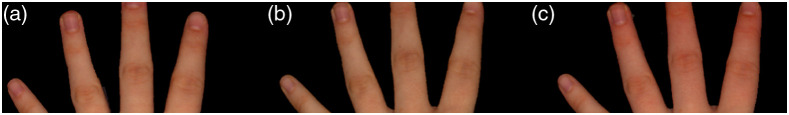
Reconstructed RGB image of human hand (a) before, (b) during, and (c) after VOT (male, 23).

RGB image, reconstructed from hyperspectral data, of the first subject (male, 23) is presented in Fig. 8. Due to the lower amount of blood during the VOT, the hand appears paler [Fig. 8(b)], while on the after image, the hand appears red [Fig. 8(c)]. The red color is due to a large amount of fresh blood, which absorbs more green and blue light. Since the presented images are not corrected for the curvature artifact, the lower intensity (shadows) are presented on the curved parts of the fingers.

**Fig. 9:**
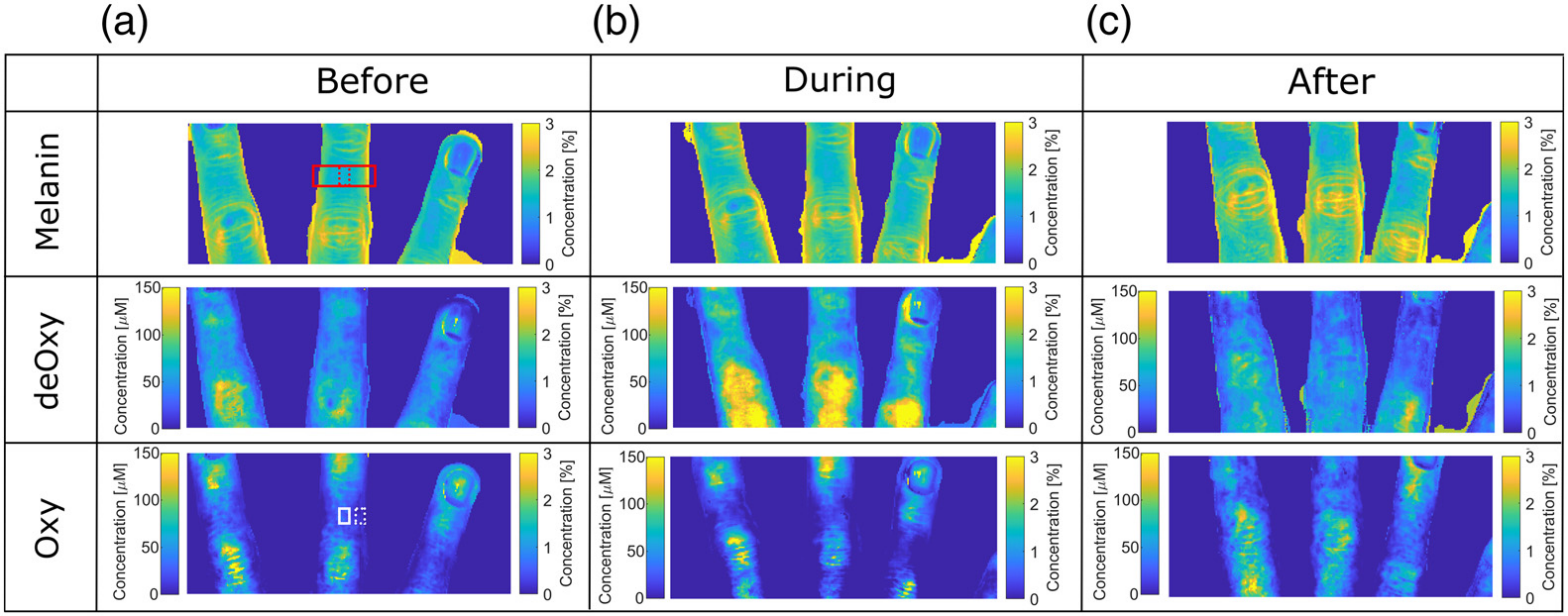
Uncorrected concentration maps of melanin, deoxyhemoglobin, and oxyhemoglobin before, during, and after VOT.

**Fig. 10:**
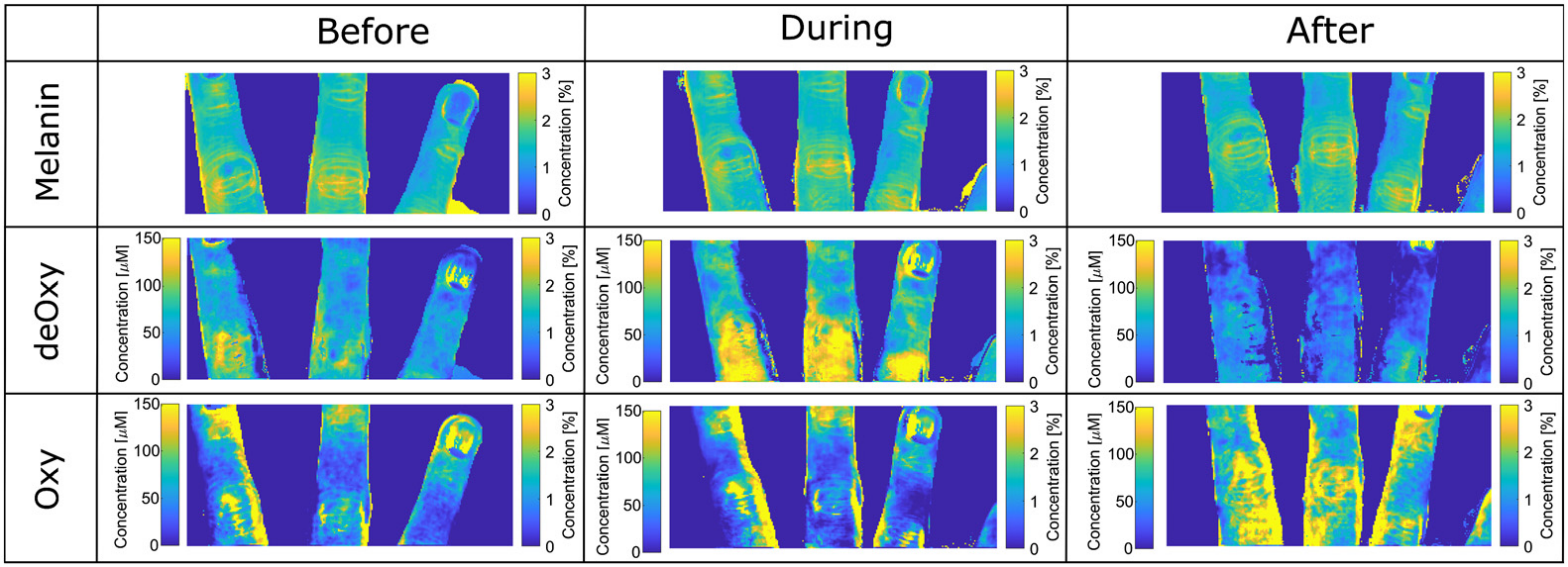
Corrected concentration maps of melanin, deoxyhemoglobin, and oxyhemoglobin before, during, and after VOT.

Adding–doubling was used to extract tissue properties from the uncorrected and corrected images. An example of the parameter distribution maps for melanin, deoxyhemoglobin, and oxyhemoglobin is shown in Figs. 9 and 10. The melanin distribution should not change during VOT because melanin is located in the epidermis and is not affected by hemodynamic changes. However, the maps extracted from the uncorrected images show an increase of melanin concentrations on the edge of the fingers. On the other hand, the decrease of melanin concentration on the finger edges of the corrected images is the consequence of a higher signal at the curved areas.

The elevated concentration regions remain in the area of the joint folds, which is due to the interreflection artifact^14^ and cannot be removed by the correction method. The interreflection artifacts are a consequence of the light reflected from the opposite sample surfaces that are also illuminating the imaged surface; thus, the affected surfaces appear to be brighter as they should be.

The deoxyhemoglobin (deOxy) maps show expected trends in uncorrected and corrected image sets. The baseline deoxyhemoglobin is relatively low; it increases during the VOT because of the oxygen consumption and blocked flow of the oxygenated blood and drops almost to zero in the after phas,e because the fresh oxygenated blood reperfuses the affected limb. However, in the uncorrected maps, the central regions of the fingers show higher deoxygenated blood concentrations, whereas the concentrations monotonically decrease at the regions closer to the finger’s boundaries. In the corrected maps, the distribution is much more uniform; the continuous decrease of the concentration by moving away from the central part is not present.

The oxygenated hemoglobin maps show the opposite trends as the deoxygenated hemoglobin maps. Here, the concentration decreases during the test and significantly increases after the cuff removal. Similar to the deoxyhemoglobin maps, the uncorrected images are affected by the artifacts in the lateral regions, whereas in the corrected images, the finger areas are much more homogeneous. Due to the inter-reflection elevated concentration regions are presented at the finger boundaries and in the skin folds. Overall, the calculated values agree well with the values found in Refs. 36 to 40.

To illustrate the curvature correction efficacy, the mean values and standard deviations of the differences between the corrected and uncorrected image parameters were calculated from the flat (solid white rectangle in left bottom corner of Fig. 9) and inclined region of the finger (dashed white rectangle in left bottom corner of Fig. 9) before VOT. The differences for the flat region were 5±0.5%, 7±0.5%, and 8%±1.1% for melanin, deoxygenated hemoglobin, and oxygenated hemoglobin, respectively. In contrast, the differences for the inclined region were significantly higher with larger standard deviations: 19±4%, 40±5%, and 75±4% for melanin, deoxygenated hemoglobin, and oxygenated hemoglobin, respectively.

**Fig. 11:**
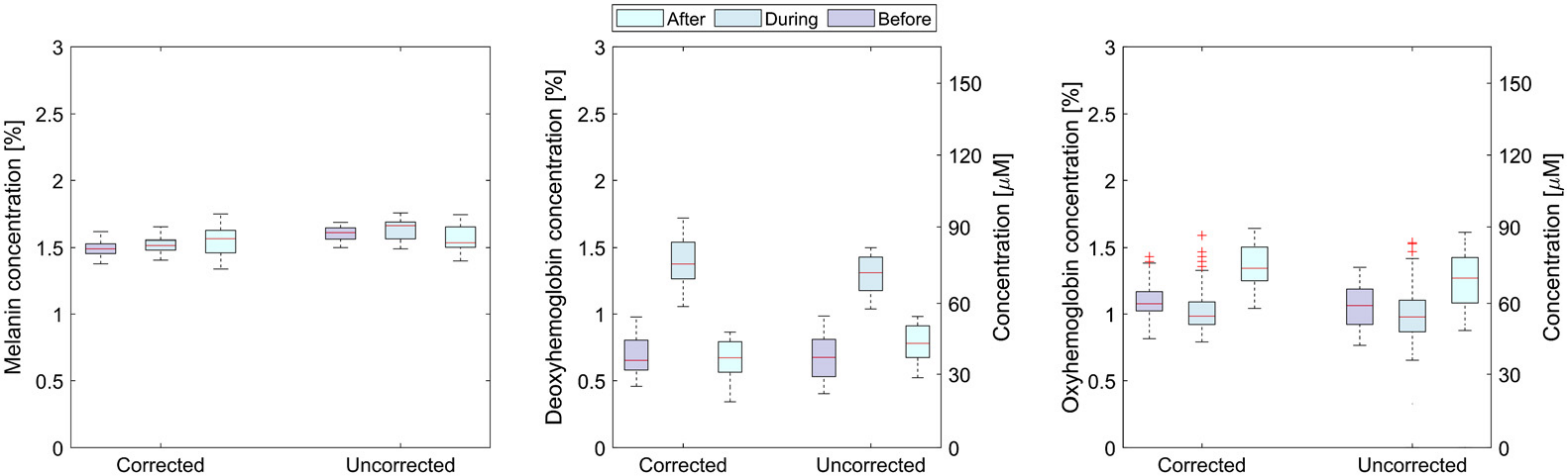
Box plots of corrected and uncorrected physiological parameters before, during, and after the VOT for the first volunteer taken on the flat part of the middle finger.

The box plots of the skin parameters obtained from the flat region (dashed red rectangle in Fig. 9) of the first volunteer’s finger (male, 23) are presented in Fig. 11. For other volunteers, results showing the same trends were obtained. The parameter values for both corrected and uncorrected images on the flat area of the finger are very similar. However, the small difference in values of the parameters obtained from the corrected images are the consequence of the height correction, which corrects the amplitude of the reflected light regarding to the sample–camera distance.

**Fig. 12:**
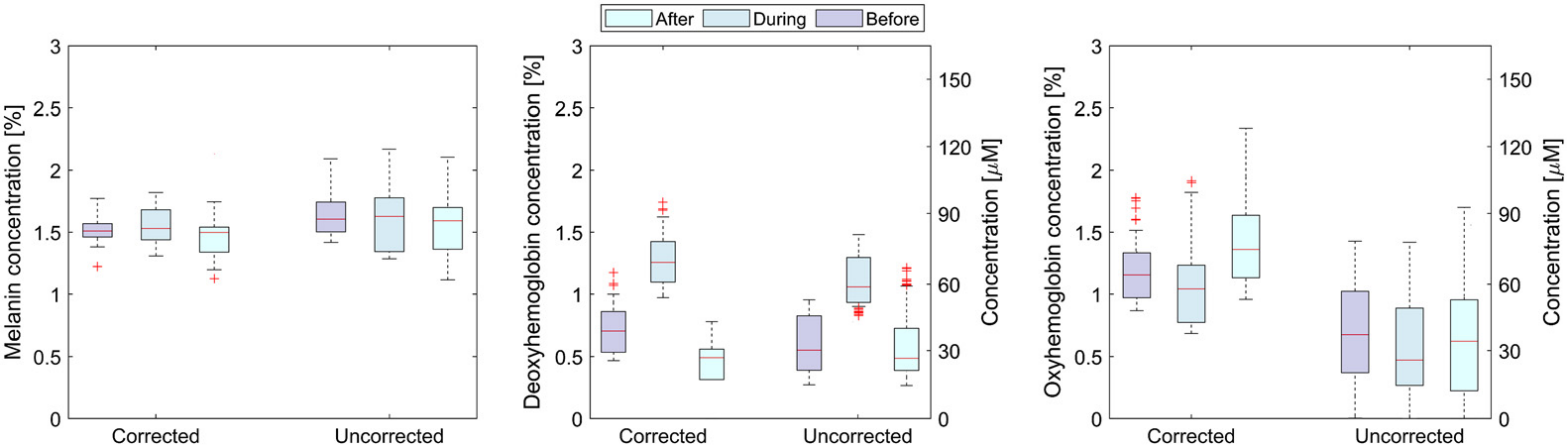
Box plots of corrected and uncorrected physiological parameters before, during, and after the VOT for the first volunteer.

Similar boxplots are presented in Fig. 12 for the part of the finger also including the curved surface (solid red rectangle in Fig. 9). The median melanin values are comparable in all phases of VOT, whereas higher values and broadening of the melanin concentration range is present in the case of the uncorrected image. The deoxygenated hemoglobin shows slightly higher median values in the corrected image, due to the increased intensity on the finger edges. The expected trend is observed in the oxygenated hemoglobin extracted from the corrected image, with a significantly lower concentrations in the uncorrected images. In contrast, the oxygenated hemoglobin concentrations are almost similar for all three phases when the uncorrected image is used, because of the inaccurate values at the finger boundary. The outliers (red crosses) on the corrected images are due to the limitations of the 3D surface extrapolation technique and Lambert cosine law on the severely inclined areas.

**Fig. 13:**
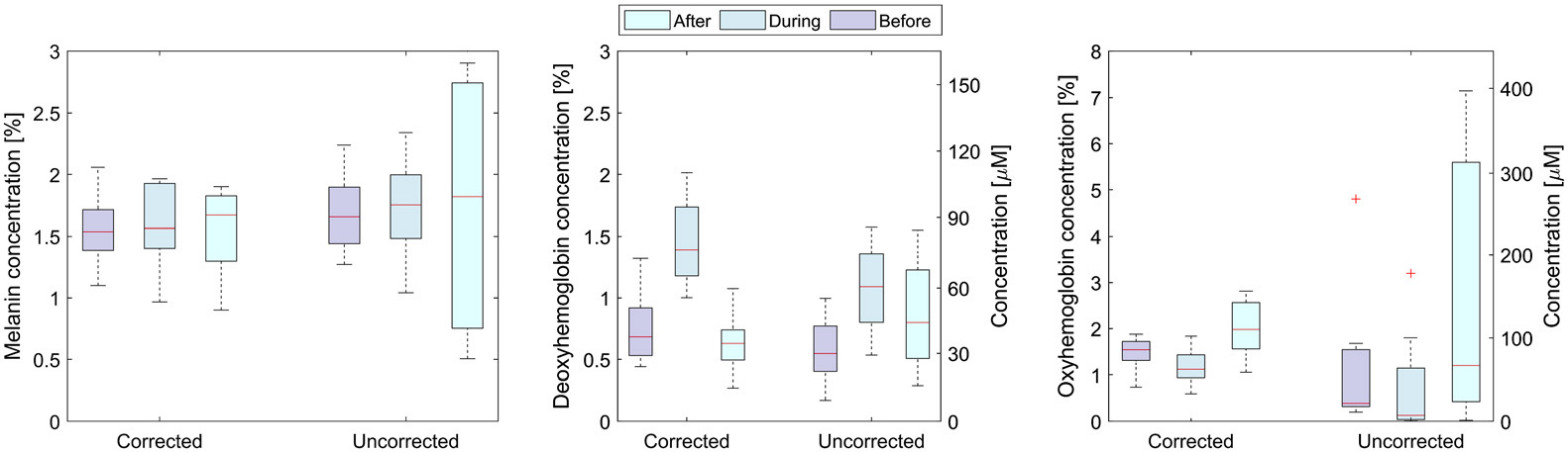
Box plots of corrected and uncorrected physiological parameters before, during, and after the VOT.

Average values of the parameters from the selected skin regions were calculated for all volunteers and presented as box plots (Fig. 13) to show the effect of image correction on a larger dataset. Here, the melanin concentrations are generally lower when the corrected image is used compared with the uncorrected image. This is expected since the curvature artifacts increase extracted absorption, as demonstrated in the tissue phantoms subsection. Similar to the first volunteer, the mean values of the melanin are comparable. Nevertheless, the uncorrected melanin concentration box for the after phase is very broad, indicating that the extracted values are not correct. The corrected deoxygenated and oxygenated hemoglobin values follow the expected trends, whereas a considerable discrepancy and broad ranges are again present in the plots for the uncorrected concentrations in the after phase.

In summary, after applying the image correction to the skin images, the extracted melanin concentrations are lower, and the distributions are similar for all VOT phases. On the other hand, the melanin values extracted from the uncorrected images are generally higher and significantly more spread in the after phase. The hemoglobin concentrations extracted from the corrected images show significant differences between different VOT phases, while these changes are reduced when the parameters are extracted from the uncorrected images. The after-phase parameters are spread over a broad value range when extracted from the uncorrected images, indicating that large blood concentrations amplify the artifacts.

## 4 Discussion

This study presents the efficacy of the 3D surface-based correction of hyperspectral data recorded by a push-broom camera, focusing especially on extracted sample parameters. The 3D surface, measured simultaneously with the hyperspectral image, was aligned with the hyperspectral image using affine transformation and used to extract surface normals. With the information about the angle between the illumination direction and surface normal, the Lambert and height corrections were applied to the hyperspectral data. The applicability of the method on image intensity for various samples was already presented in Ref. 14. In this research, the effect of the correction on the reflectance spectra and extracted parameters is presented.

The development of Siliglass tissue phantoms was based on a modified recipe by Sekar et al.,^19^ using the same phantom labels. The phantoms were hemispherical and covered inclination angles from 0 deg to 65 deg. During the manufacturing of the phantoms, the absorber and scatterer concentrations vary due to the compound measurement errors and accumulation of the material on the edge of the cup. Therefore, after every part of the procedure, the mixture was carefully weighed, and more reliable concentrations were calculated. The maximum difference between the concentrations provided by Sekar et al.^19^ and calculated as 0.7% and 0.2% for absorber and microspheres, respectively. Thus, the phantoms prepared in this study agree well with the original phantoms.

Seven different phantoms were created and imaged with the pushbroom hyperspectral system. Their images showed that higher inclination angles resulted in lower, inaccurate reflectance spectra. After applying the correction, the reflectance became significantly more homogeneous. More critical than the correction effect on the spectra is its impact on the extracted sample parameters. In our research, a one-layer IAD method was used to extract the parameters. Before applying IAD to the whole images, the parameters extracted from the central, relatively flat areas were compared with those determined from the phantom preparation data. The relative difference of 2% to 8% and 5% to 12% is present for $C_{\mathrm{abs}}$ and $C_{\mathrm{sc}}$. Considering the standard deviations of the extracted parameters and the phantom preparation data, it is evident that the extracted values are well within the error intervals.

In Fig. 5, the concentration maps for the corrected and uncorrected phantom images are presented. The corrected images are homogeneous over the whole phantom region, except for discrepancies occurring on the edges. These discrepancies result from the large inclination angles where profilometry is not reliable, and Lambert correction does not perform adequately. The maximum inclination angles were up to 60 deg. On the contrary, in the uncorrected maps, the homogeneous area is very small (up to the inclination angle of 20 deg). On the edges, IAD returns maximum allowed values for specific parameters (i.e., boundary values) indicating inadequate fits.

Average $C_{\mathrm{abs}}$ and $C_{\mathrm{sc}}$ were calculated from the concentration maps. The area for the analysis was limited by the maximum angle of corrected images given in Table 4. In general, the average of $C_{\mathrm{abs}}$ is higher for the uncorrected images since the algorithm predicts higher absorption on the inclined areas due to the underestimated reflectance in these areas. The exact opposite effect is observed for $C_{\mathrm{sc}}$. The corrected images have approximately 10 times lower STD values for both $C_{\mathrm{abs}}$ and $C_{\mathrm{sc}}$, because the parameter maps are significantly more homogeneous. Specifically, the average STD of the corrected $C_{\mathrm{abs}}$ and $C_{\mathrm{sc}}$ is 0.19% and 0.06%, respectively, whereas the corresponding average STD values for the uncorrected images are 2.00% and 0.57%.

The maximum inclination angles up to which the correction is effective were calculated from the phantom results (Table 4). In our previous article,^14^ we found that the correction works up to 70 deg at 650 nm if only image intensity is considered. However, when parameters are extracted from an image, the correction works up to 60 deg inclination angle. The phantom imaging results also show that the correction is the most effective for the phantoms with lower concertation of absorber and scatterer, where the maximum inclination angle increases almost six times.

The same imaging and imaging analysis procedure were repeated for human hands. To estimate the correction effect on the extracted physiological parameters, the hands were imaged before, during, and after VOT. The average extracted values of the physiological parameters are comparable to those found in the literature. The average extracted melanin concentration for all volunteers is 1.54±0.8%, which coincides well with Verdel et al.,^34^ who reported 1.4±0.1%. The average oxygen saturation was 46±8% during and 77±10% after the test, which coincides with ranges reported by Fredriksson et al.^41^ and Bruins et al.^42^

Figure 12 shows similar melanin concentration for all test phases, smaller dispersion of deoxyhemoglobin concentration, and a lower percentage of oxyhemoglobin concentrations at the minimum values, when corrected images are used. However, a higher number of outliers are present, which are the points in the areas with large inclinations, where Lambert cosine law and 3DP are not accurate. From Fig. 13, it is possible to assess the effects of the correction on physiological parameters over the whole group of volunteers. The average melanin concentration before, during, and after VOT, calculated from the corrected images, is 1.57±0.36%, whereas the average calculated from the uncorrected images is 1.75±0.72%. The latter average has approximately two times larger standard deviation due to the larger spread of the extracted concentrations and less accurate fit. For the deoxyhemoglobin concentration, the expected concentration increase during and drop after the test is more pronounced in the corrected data. The deoxyhemoglobin increase is 49% higher in comparison to the uncorrected images. The corrected data correspond more closely to Ref. 43, where relative oxygen saturation on hands during VOT drops for around 50% after 150 s. On our images, the average oxygen saturation drops for 40±10% on corrected and 66±12% on uncorrected images.

When showing the extracted parameters in the box plots, the difference between the corrected and the uncorrected parameters is somewhat masked (Figs. 12 and 13). The reason is that the box plots include the values from the regions, where the effect of the correction is small (flat regions) and the values from the regions where the correction effect is significant (the curved areas). Thus, the mean value can be very similar, but the spread of the values is much larger in the case of the uncorrected images. For more direct comparison, the parameter values in the flat and inclined regions should be compared separately, as it was done in the case regions marked with the white rectangles shown in Fig. 9. Therefore, the correction effect is effectively even larger than presented in the box plots.

Successful implementation of the correction method was already reported by Gioux et al.,^9^^,^^12^ when SFDI was used. The authors demonstrated that the correction method could be used for surfaces with inclinations up to 40 deg, which is <47  deg to 62 deg found using our approach. The method was tested on hemispherical phantoms, but only for one case of $C_{\mathrm{abs}}$ and $C_{\mathrm{sc}}$; thus, a thorough assessment of the method for different concentrations of absorbers or scatterers could not be performed. The positive effect of the correction was also presented for a human finger but just the reduction of STD of extracted absorption and scattering coefficients.

Zhao et al.^10^ demonstrated that the correction could be functional up to 70 deg of inclination when modified Lambert law and SFDI are used. Solid phantoms with different absorption and scattering coefficients were studied, but the correction effect is presented for one tissue phantom only. The main disadvantage of their technique is its dependence on the presence of flat areas on samples. The method searches for a correction coefficient k to minimize the difference between the signal on the flat and curved areas, assuming homogeneous sample. Therefore, the method does not work for samples with heterogeneous optical properties and those without flat surface areas.

Although we included seven different tissue phantoms in this study, trying to cover the typical tissue optical parameters, the correction method could be more extensively tested by including additional phantoms with higher variability of optical parameters. Therefore, the impact of optical parameters on algorithm performance could be thoroughly explored and better guidelines for imaging different tissues provided. According to Table 4 and Refs. 22 and 44, the maximum inclination angles for different tissues regarding their absorption and scattering coefficients would be above 51 deg for Caucasian skin, above 56 deg for muscle, and around 47 deg for bone.

Currently, the presented correction method works for angles up to ∼60  deg, depending on tissue optical properties. The maximum angle could be increased by including modification of Lambert’s law, as Zhao et al.^10^ suggested. Moreover, the profilometry module could be upgraded with additional line lasers to more accurately measure higher inclinations and eliminate the areas without laser line illumination. Different polarizers should be implemented to cover the larger spectral range. Here, only spectral region 430 to 750 nm was used, although the systems provide information for the 400- to 1000-nm spectral region. The infrared region contains valuable information about additional chromophores (e.g., water and lipids) and has a larger penetration depth. To analyze this additional spectral region, IAD should be extended by including these additional chromophores, scattering coefficient ansatz with both Mie and Rayleigh ansatz, and adding additional skin layers. Using the extended IAD model, we could study the effect of the correction algorithm also in the infrared spectral range.

## 5 Conclusions

This article demonstrates that the reliability of reflectance spectra measured with a pushbroom hyperspectral camera and extracted tissue parameters is significantly improved when the 3D surface-based curvature and height correction method is used. The corrected reflectance images allow a more trustworthy analysis of not only flat but also highly inclined areas. The method was successfully applied on images of tissue phantoms of known optical properties and geometry and VOT images of human hands. The results show good agreement between the expected and extracted parameters, when the corrected images are used. The proposed method could be applied to any reflectance imaging system and should be used whenever quantitatively accurate parameters need to be extracted from the images of a curved sample.

## 6 Appendix A

**Fig. 14:**
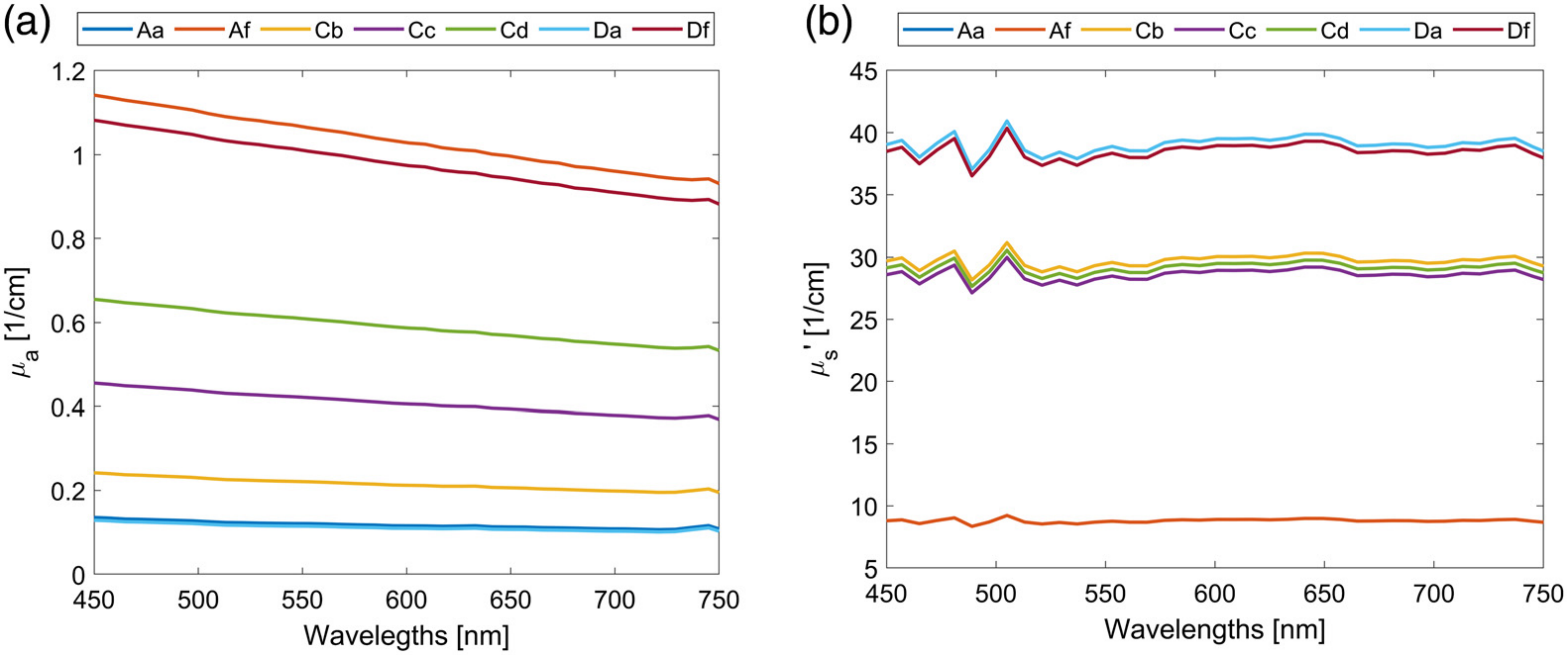
(a) Absorption and (b) reduced scattering coefficient spectra of the prepared phantoms.

Absorption and reduced scattering coefficients of the phantoms were calculated using the corresponding absorber and scatterer concentrations (Tables 2 and 3), and Eqs. (8), (9), ${\mu_{s}}^{\prime }=\mu_{s}(1-g)$ (see Fig. 14).

## Code, Data, and Materials Availability

There is no code essential for reproducibility or supplementary data available for this manuscript.
